# Mesenteric Cystic Lymphangioma Causing Small Bowel Obstruction in a Neonate

**DOI:** 10.7759/cureus.91130

**Published:** 2025-08-27

**Authors:** Gregory Stimac, Federico G Seifarth, Joseph D Drews, Patrick Bacaj, Lea A Wehrli

**Affiliations:** 1 Surgery, West Virginia University School of Medicine, Morgantown, USA; 2 Pediatric Surgery, West Virginia University School of Medicine, Morgantown, USA; 3 Pathology, West Virginia University School of Medicine, Morgantown, USA

**Keywords:** bowel obstruction, case report, cystic lymphangioma, mesenteric lymphangioma, mesenteric lymphovascular malformation, pediatric surgery, pediatric volvulus, volvulus

## Abstract

Mesenteric cystic lymphangiomas (MCLs) are benign malformations of the lymphatic system that are most commonly diagnosed during childhood. The range of presentation varies with anatomic location. MCLs of the gastrointestinal tract can lead to bowel obstruction, and surgical intervention is warranted for those presenting in the acute setting. We report a case of a female neonate who was brought to the emergency department with a history of 24 hours of bilious emesis and abdominal distention. An abdominal X-ray was suspicious for bowel obstruction, and the patient was emergently taken to the operating room. A cystic lesion was identified in the mesentery of the jejunum, causing volvulus and bowel obstruction. The mass was resected, including the adjacent bowel. Pathology revealed a macrocystic lymphatic malformation (lymphangioma). The patient required total parenteral nutrition in the acute postoperative phase and has since weaned off successfully. The patient was ultimately discharged after an uneventful hospital course and was doing well at her 12-month follow-up. We concluded that MCLs are benign malformations that can be resected at the time of surgery. Due to the benign nature of these lesions, outcomes and prognosis are excellent.

## Introduction

Mesenteric cystic lymphangiomas (MCLs), also known as lymphovascular malformation, are rare benign malformations of the lymphatic system located within the bowel mesentery. Most commonly, lymphangiomas are found within the head and neck area, and in less than 5% within the bowel mesentery. The small bowel mesentery is more frequently affected than the mesocolon [1]. The incidence of abdominal lymphatic malformations in patients less than 10 years of age was described to be 1:20,000-1:20,200, with a slight male predominance [2-4]. In 10% the diagnosis has been made prenatally, while the majority is diagnosed within the first five years of life [3-5]. Even though 65% are present at birth, there is a paucity of literature describing MCL in neonates [5-7]. The clinical manifestation of MCLs is heterogeneous. Patients remain asymptomatic, with tumors often discovered incidentally during imaging studies or at the time of laparotomy for other surgical indications. Symptomatic individuals present with a spectrum of clinical features, depending on the location and size of the lesion within the peritoneal cavity. Symptoms range from vague abdominal discomfort and distension to more acute settings such as small bowel obstruction necessitating urgent surgical intervention [7,8]. Management of MCLs involves surgical resection. The benign nature of the MCL generally results in a favorable outcome and excellent long-term prognosis after surgical intervention [1,3]. This manuscript was prepared following the CARE guidelines (https://www.care-statement.org).

## Case presentation

**Figure 1:**
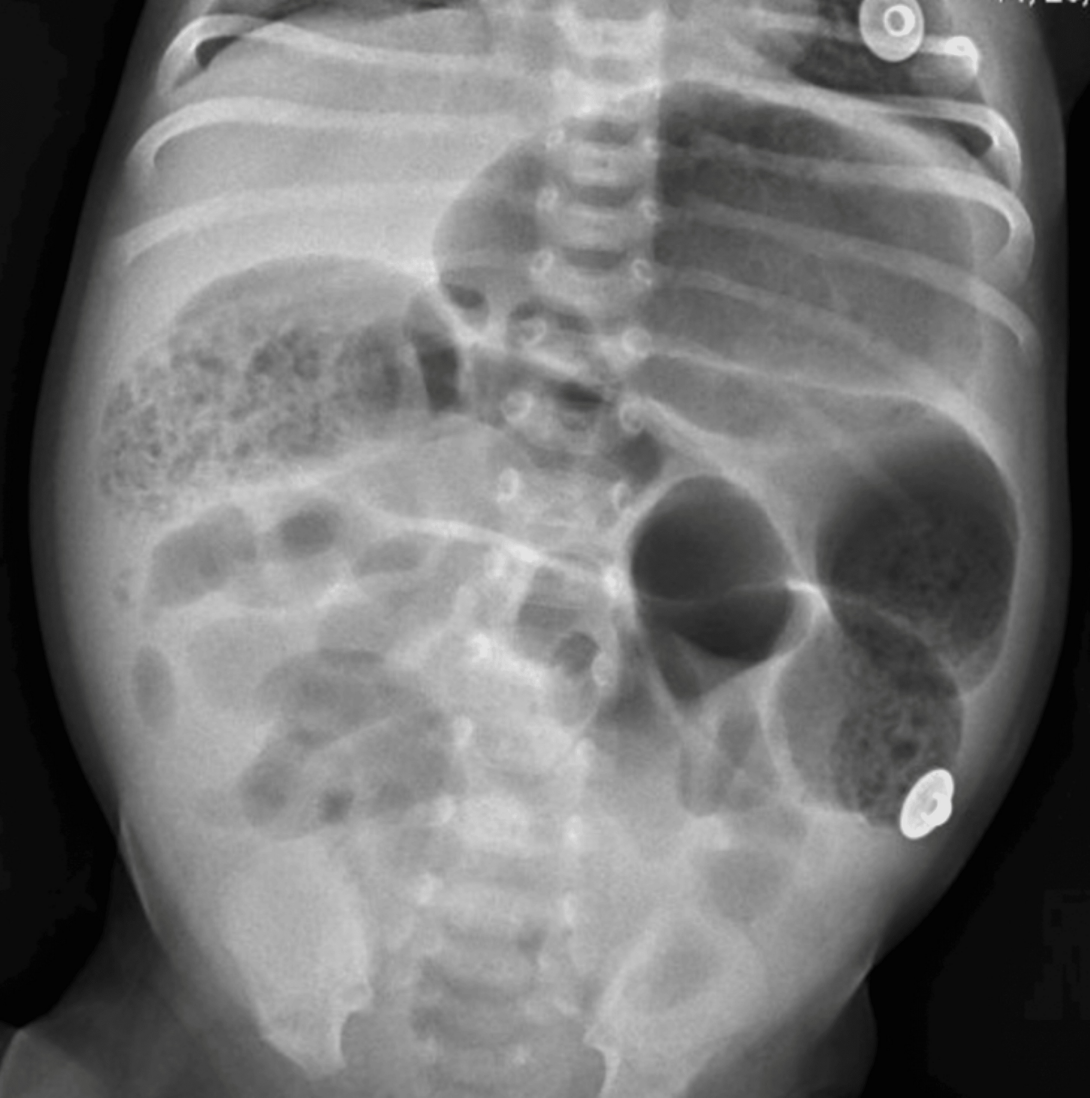
Abdominal radiograph at initial presentation Abdominal radiograph demonstrating findings suggestive of bowel ischemia or small bowel obstruction. The bowel pattern is irregular with gaseous distention of the stomach and distention of multiple loops of bowel with mottled lucencies. There is no portal venous gas.

A 27-day-old white female neonate presented to the emergency department with abdominal distention for approximately 24 hours. The patient initially presented to an outside hospital with bilious emesis, hematochezia, jaundice, and mottled extremities and was immediately transferred to our tertiary care facility. Vital assessment demonstrated tachycardia to 190 beats per minute and hypotension to 72/36 mmHg with a respiratory rate of 52 breaths per minute. The patient was immediately started on intravenous crystalloid fluid resuscitation and broad-spectrum antibiotics (ampicillin, gentamicin, and metronidazole). A nasogastric tube was placed, which drained a large volume of bilious enteric content. Laboratory studies were significant for a leukocytosis of 15.8 × 103 /µL, lactate dehydrogenase of 4.7 mmol/L, creatinine of 0.51 mg/dL, and an abdominal film demonstrated gaseous distention of the stomach with small bowel dilation and mottled lucencies (Figure 1).

Pediatric surgery was immediately consulted due to concern for intestinal obstruction. The physical exam demonstrated a firm and distended abdomen with cyanotic discoloration. Due to concern for a small bowel volvulus with obstruction, the patient was taken emergently to the operating theatre for an exploratory laparotomy. The patient was otherwise healthy without any associated medical, surgical, or family history.

**Figure 2:**
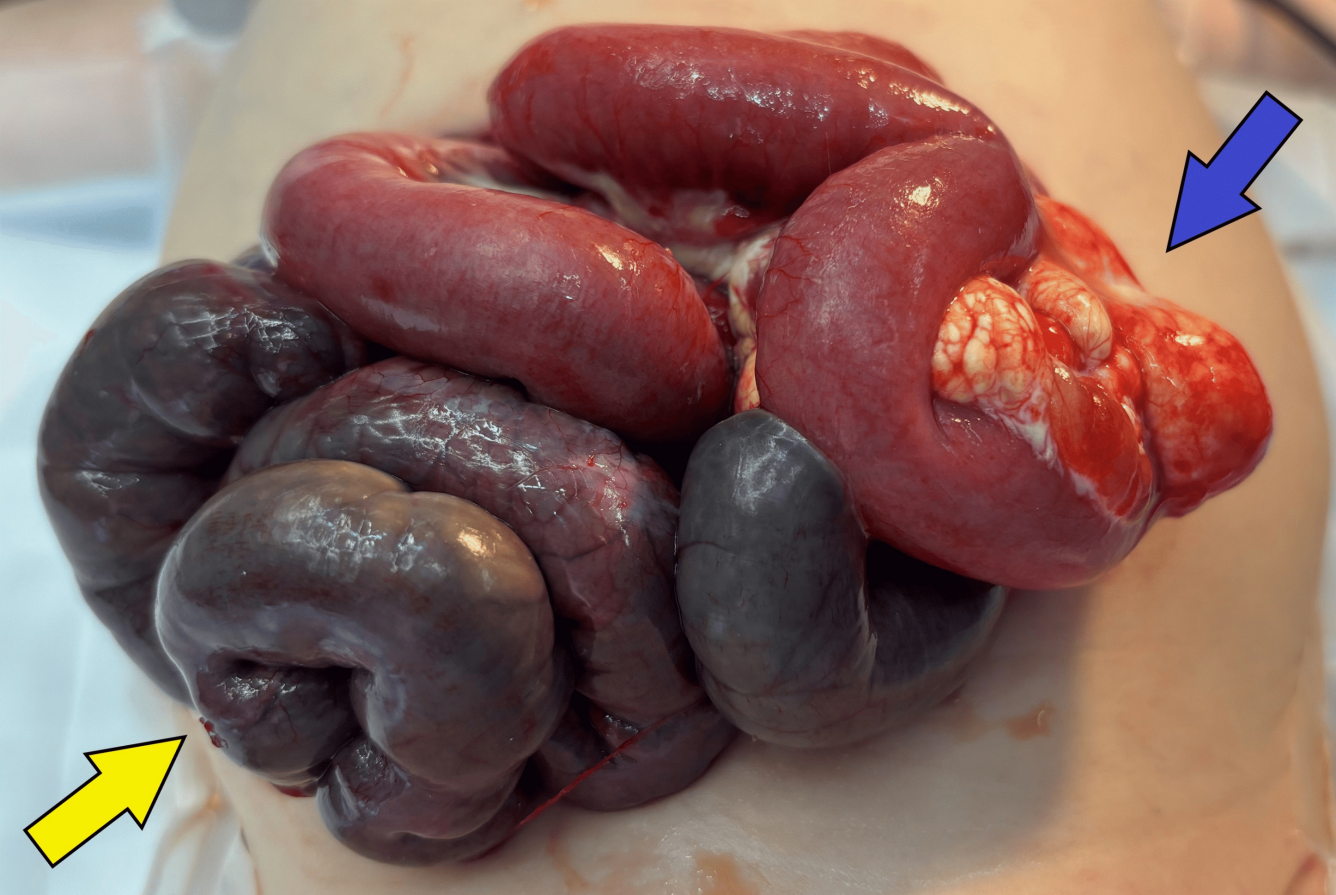
Intraoperative findings - part 1 Intraoperative photograph illustrating frank bowel ischemia caused by the mesenteric cystic lymphangioma (MCL). Exploratory laparotomy revealed a cystic lymphangioma (blue arrow) in the mesentery of the jejunum. This image illustrates the section of small intestine affected by ischemia due to volvulus by the MCL acting as an anchor point. Following detorsion, there was persistence of necrosis, as pictured (yellow arrow). The necrotic jejunum was resected during the initial laparotomy, and additional small bowel was allowed to demarcate on subsequent takebacks to the operating room prior to formal resection of the MCL.

**Figure 3:**
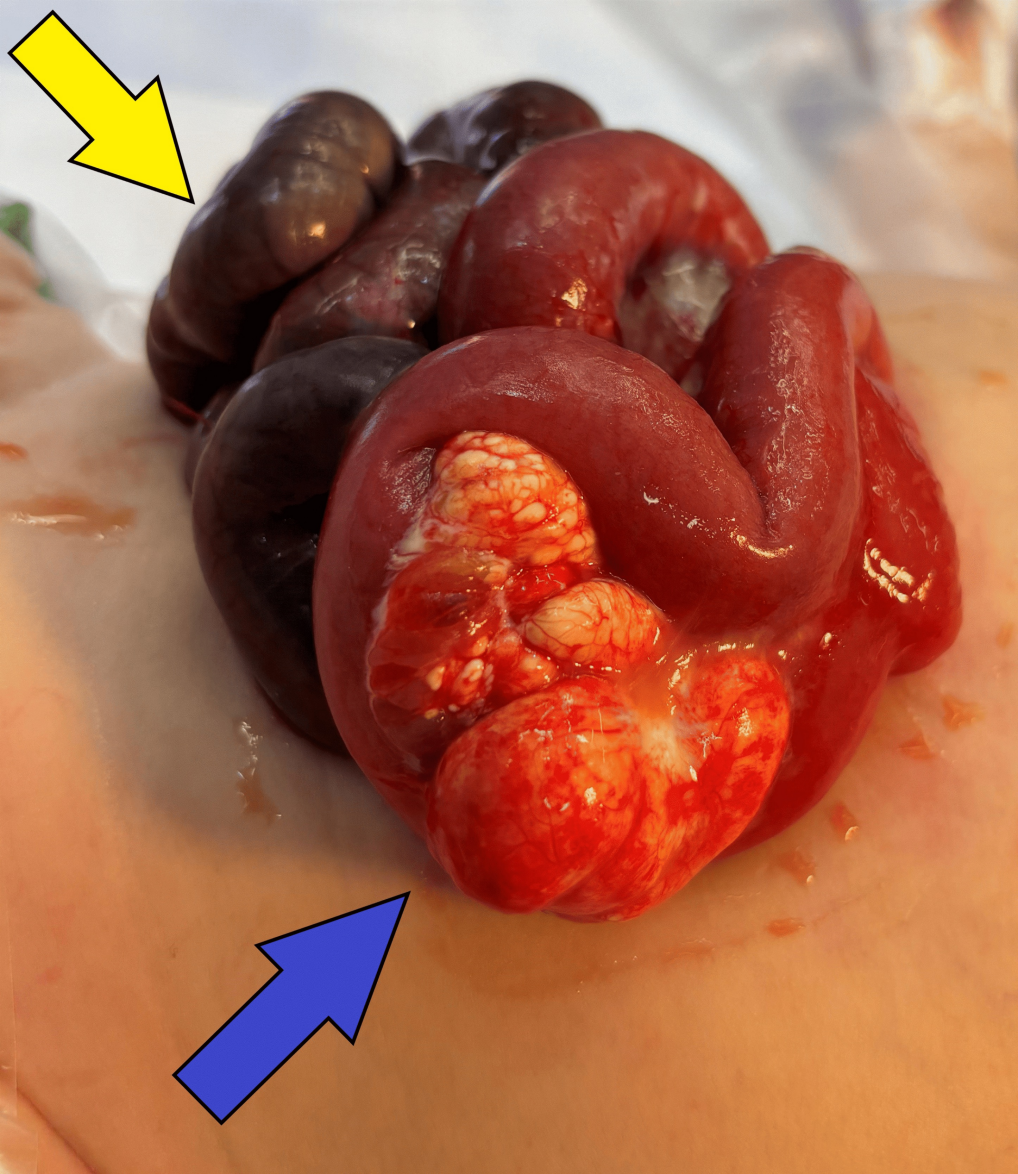
Interoperative findings - part 2 Intraoperative photograph showing the mesenteric cystic lymphangioma (MCL) (blue arrow) with cystic lesions located within the mesentery. The necrotic jejunum is marked with a yellow arrow.

The patient underwent an exploratory laparotomy, which revealed a 360-degree segmental volvulus of the small bowel with ischemia due to an MCL located 36 cm distal to the ligament of Treitz (Figures 2-3).

The cyst served as an anchoring point, predisposing the intestinal loops to undergo torsion about the mesenteric axis. A 30-cm necrotic jejunum was resected; an additional 20 cm of small bowel had questionable viability. Temporary closure and a second-look operation were decided prior to definitive anastomosis to ensure viable bowel at the time of final closure. A silo was placed and wrapped in Vaseline gauze (Covidien, Mansfield, MA) and Kerlix (Medline, Northfield, IL). The patient was then transported to the pediatric intensive care unit (PICU) in stable condition, and total parenteral nutrition (TPN) was initiated. The patient was taken back to the operating theater within 12 hours to reassess the small bowel. On takeback, 90 cm of the distal ileum was necrotic, which was resected. The ileocecal valve was well-perfused. A decision was made to replace the silo due to persistent edema and questionable perfusion of the remaining small bowel. A second takeback was performed after 48 hours from the initial operation, during which the mesenteric cyst was excised. An end-to-end, hand-sewn anastomosis was performed, connecting 46 cm of proximal jejunum from the ligament of Treitz to 4 cm of terminal ileum from the ileocecal valve. A 12 French, 1.2 cm button gastrostomy tube was then placed, and the abdomen was closed.

**Figure 4:**
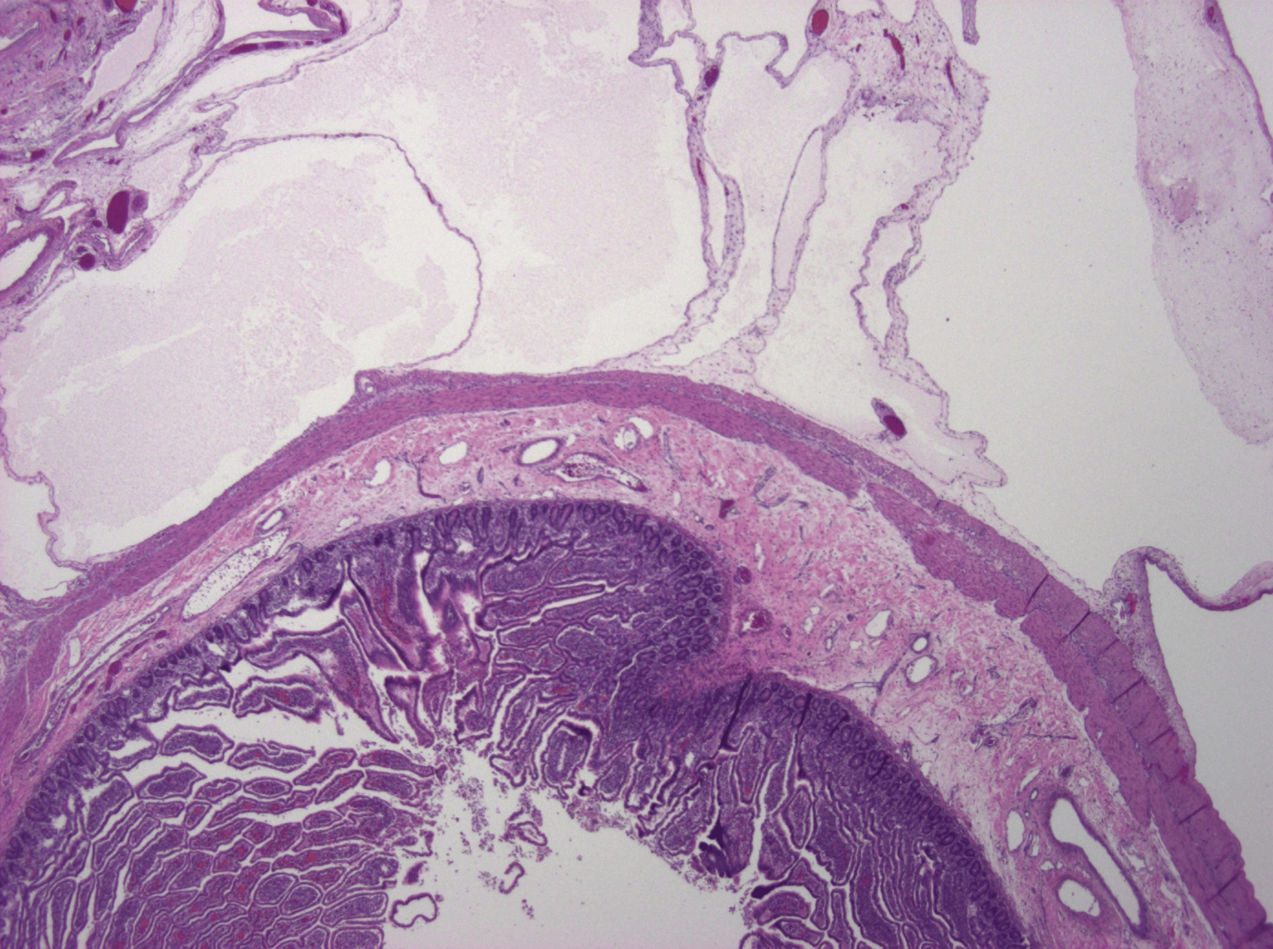
Mesenteric lymphangioma at low magnification (20×, H&E stain) A full-thickness section of small bowel occupies the lower half of the image. Above it, variably-sized microcysts replace the mesenteric fat. The microcysts display thin walls and contain pale proteinaceous fluid.

**Figure 5:**
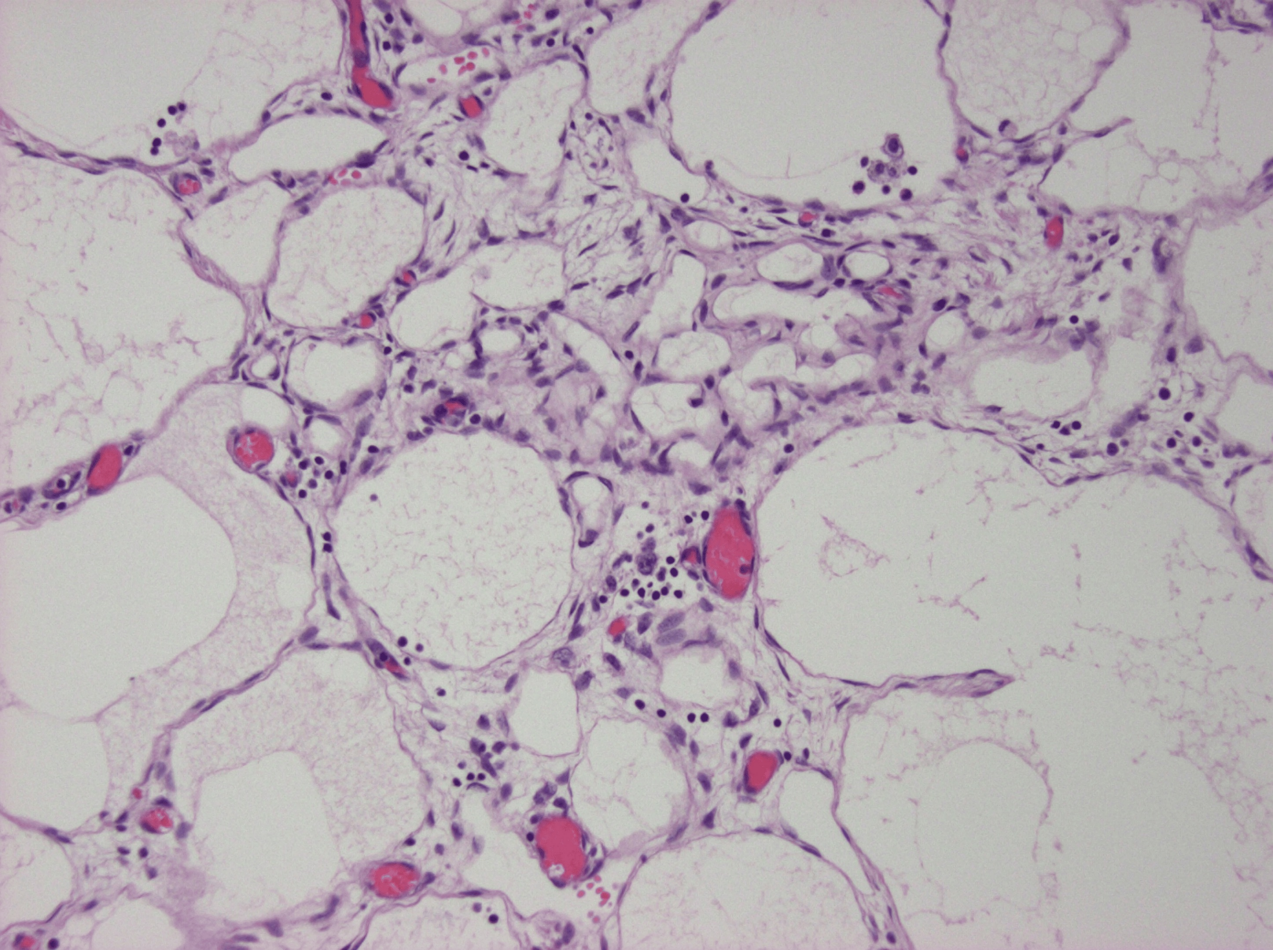
Mesenteric lymphangioma at high magnification (200×, H&E stain) Ectatic lymphatic vessels lined by flattened endothelial cells form the microcysts. The vessel walls lack smooth muscle. The scant supporting stroma is edematous and contains scattered lymphocytes. The features are characteristic of a lymphangioma.

The patient’s postoperative course was unremarkable. Trickle tube feeds were started when bowel function returned on postoperative day four from definitive closure. The TPN was slowly weaned after the patient demonstrated weight stability on continuous enteral nutrition. The patient was discharged after a 36-day hospital course. At one-month follow-up, the patient was growing appropriately, and enteral nutrition was changed to initially cycled feeds and then weaned over six months. On her 12-month follow-up, she was doing very well and growing along the 30th weight percentile. Histopathology confirmed an MCL measuring 5.0 × 4.5 × 3.0 cm with fibrinous exudate, and sections through the cyst revealed multiple ectatic, thin-walled vessels of varying sizes containing proteinaceous fluid (Figures 4-5).

## Discussion

Here, we detail a case of an MCL in a neonate causing a segmental small bowel volvulus leading to complete bowel obstruction and ischemia, which required emergent operative intervention. MCLs are generally diagnosed during early childhood within the first five years of life [4,5], with a third presenting due to bowel obstruction and volvulus [9]. To date, case reports of neonates presenting with obstruction and/or volvulus due to mesenteric lymphangioma are rare. Lymphovascular malformations are most commonly found in the neck (75%) or axillae (20%), and are rarely located in the bowel mesentery [1]. Clinical presentations of MCLs of the abdomen can be diverse, with abdominal pain being the leading symptom, followed by abdominal mass [4]. Approximately 30-50% of MCLs present as bowel obstruction from mass effect on adjacent bowel, adhesions, or volvulus [8-10]. Asymptomatic lesions had been either diagnosed prenatally or often serendipitously identified during imaging performed for evaluation of other conditions [1,3,4,11]. Final diagnosis is established intraoperatively and confirmed postoperatively through histopathology and immunochemistry. Histopathology demonstrates mesothelial cells reactive to cytokeratin, and Prox1 and CD31 staining are the most reliable characteristics [11].

Awareness of MCLs is crucial, given their benign nature, because surgical resection is the treatment of choice and is usually curative. Troum and Solis [8] described a case of MCL causing bowel obstruction in a patient with cerebral palsy, managed by resection and primary anastomosis. The child made an uneventful recovery and had no evidence of recurrence after a six-month follow-up [8]. Weeda et al. documented two cases with MCL of the small bowel, both addressed with resection and primary anastomosis [7]. Abdulraheem et al. described excision of a symptomatic descending mesocolic MCL in a patient whose postoperative course was unremarkable [12]. Upon review of the literature, neonates are typically diagnosed prenatally [1,3,4]. The most extensive series is reported by Steyaert et al. in 1996, who presented a case series of 21 patients over a time period of 20 years, all treated by enucleation or resection [13]. 

Imaging plays a crucial role in diagnosis when patients are being evaluated in the outpatient setting. Evaluation usually begins with a plan film or ultrasound study. If these are inconclusive, CT imaging typically demonstrates a thin-walled, multiseptated cystic mass with Hounsfield units varying from fluid to fat consistency [14]. MRI can also be used and is more specific in determining the cystic contents [15]. Surgical resection of MCLs is required even if asymptomatic due to the risk of volvulus or obstruction [4,16]. Recurrence is low if complete resection has been performed with excellent prognosis [1].

## Conclusions

MCLs, although rare, can be a cause of bowel obstruction in the pediatric population. Surgical resection is typically curative and should be pursued even in asymptomatic individuals with an excellent prognosis.
